# The Craving-Manager smartphone app designed to diagnose substance use/addictive disorders, and manage craving and individual predictors of relapse: a study protocol for a multicenter randomized controlled trial

**DOI:** 10.3389/fpsyt.2023.1143167

**Published:** 2023-05-15

**Authors:** Fuschia Serre, Sarah Moriceau, Léa Donnadieu, Camille Forcier, Hélène Garnier, Jean-Marc Alexandre, Lucile Dupuy, Pierre Philip, Yannick Levavasseur, Etienne De Sevin, Marc Auriacombe, for Craving-Manager RCT investigator group

**Affiliations:** ^1^University of Bordeaux, Bordeaux, France; ^2^SANPSY, UMR 6033, CNRS, Bordeaux, France; ^3^Pôle Inter-établissement d’Addictologie, CH Ch. Perrens and CHU de Bordeaux, Bordeaux, France

**Keywords:** craving, addiction, ecological momentary intervention, randomized controlled trial, cognitive-behavioral therapy, mobile phone apps, mHealth, substance use disorders

## Abstract

**Background:**

The rate of individuals with addiction who are currently treated are low, and this can be explained by barriers such as stigma, desire to cope alone, and difficulty to access treatment. These barriers could be overcome by mobile technologies. EMI (Ecological Momentary Intervention) is a treatment procedure characterized by the delivery of interventions (messages on smartphones) to people in their daily lives. EMI presents opportunities for treatments to be available to people during times and in situations when they are most needed. Craving is a strong predictor of relapse and a key target for addiction treatment. Studies using Ecological Momentary Assessment (EMA) method have revealed that, in daily life, person-specific cues could precipitate craving, that in turn, is associated with a higher probability to report substance use and relapse in the following hours. Assessment and management of these specific situations in daily life could help to decrease addictive use and avoid relapse. The Craving-Manager smartphone app has been designed to diagnose addictive disorders, and assess and manage craving as well as individual predictors of use/relapse. It delivers specific and individualized interventions (counseling messages) composed of evidence-based addiction treatments approaches (cognitive behavioral therapy and mindfulness). The Craving-Manager app can be used for any addiction (substance or behavior). The objective of this protocol is to evaluate the efficacy of the Craving-Manager app in decreasing use (of primary substance(s)/addictive behavior(s)) over 4 weeks, among individuals on a waiting list for outpatient addiction treatment.

**Methods/design:**

This multicenter double-blind randomized controlled trial (RCT) will compare two parallel groups: experimental group (full interventional version of the app, 4 weeks, EMA + EMI), versus control group (restricted version of the app, 4 weeks, only EMA). Two hundred and seventy-four participants will be recruited in 6 addiction treatment centers in France.

**Discussion:**

This RCT will provide indication on how the Craving-Manager app will reduce addictive use (e.g., better craving management, better stimulus control) in both substance and behavioral addictions. If its efficacy is confirmed, the app could offer the possibility of an easy to use and personalized intervention accessible to the greatest number of individuals with addiction.

**Clinical Trial Registration:**

ClinicalTrials.gov: NCT04732676.

## 1. Introduction

Substance use disorders and addictive non-substance disorders (that we combine under the term addiction) are the most prevalent psychiatric disorders in the general population (1–4) and are associated with significant medical and social harm (5). Addiction is defined as a loss of or reduced control of use of a reinforcer (substances or behaviors) that translates into continued use despite its harmful consequences (6, 7). Addiction may be considered a chronic disease (8, 9), and relapse is the major clinical outcome and the focus of treatments (10). Contemporary definitions of addiction emphasize on the inability to limit use despite the clearly negative consequences, as well as the overwhelming desire to use, also named “craving” or “pathological craving” (7). Craving is an unwanted, persistent, and intense desire to use the substance, or engage in a behavior. It is currently considered a central component in several addiction models (11, 12) and was introduced as a diagnostic criterion in the Diagnostic and Statistical Manual of Mental Disorders, Fifth Edition–DSM-5 (6, 7, 13, 14). Craving can be considered as a complex subjective phenomenon strongly influenced by internal factors and environmental context and its expression fluctuates, in duration and intensity, within hours (15, 16).

The major role of craving in relapse has been highlighted by numerous studies for both substance and non-substance addictions (17, 18). Based on such research, craving is currently considered a major treatment target (7, 19). Some factors may contribute to induce craving, and therefore increase the risk for relapse. In particular, studies have experimentally demonstrated the ability of certain stimuli (cues) previously associated with substance use (such as syringes, bottle, smoke, etc.) to induce physiological responses and to increase craving in the absence of the substance (20–23) as well as the activation of brain reward system (24, 25). Similar responses have also been demonstrated for gambling (26, 27) and food addiction (28). These responses, also referred to as “cue reactivity,” are remarkably stable and are specifically induced by stimuli associated with substance use but not by neutral stimuli (20). This reactivity seems to persist long after cessation of use (29, 30). Although the relationship between these phenomena and relapse has long been controversial, laboratory data showed a link between the intensity of the craving episode induced after exposure to these stimuli, and relapse for tobacco, alcohol, cocaine and heroin use (22, 31–35).

In addition to these so-called “standard” cues, linked to the substance itself or related paraphernalia (i.e., equipment necessary for substance use), there are also more complex and varied stimuli, specific to each subject’s personal history and substance use habits. These “person-specific” cues correspond to a variety of experiences, contexts, environments or emotions, associated with addictive use and specific to each subject. Exposure to these person-specific cues is associated with higher levels of craving than exposure to standard stimuli, both in laboratory (36), and in individuals’ natural environment (37). Ecological Momentary Assessment (EMA), a method that uses mobile technologies for collecting real-time data in daily life (38), has confirmed the predictive nature of exposure to these conditioned stimuli on craving intensity and later substance use over the following hours, regardless of the type of substance or behavior considered (21, 37, 39, 40). In addition, analyses showed that most subjects remained exposed to these person-specific stimuli during the first weeks of treatment, in a more pronounced way than stimuli directly related to substances that could be easier to avoid (37, 39).

Treatment of substance use disorders/behavioral addictions aims to reach and maintain abstinence, or at least a significant reduction in addictive use, by avoiding or controlling high-risk situations for relapse. As a major predictive factor for relapse, craving management can be an important target for pharmacological and psychosocial treatments developed in this field (41). Psychotherapies, in particular, cognitive-behavioral therapies (CBT), as part of a comprehensive approach to addiction treatment, are pragmatic interventions allowing evaluation and awareness of the mechanisms involved in recovery and relapse (42). CBTs aim to help the subject to analyze the usual contexts and emotions related to addictive use (exposure to stimuli, proposal of use by a peer, conflict situation, etc.) and to identify high-risk situations for relapse. CBTs then provide cognitive and behavioral strategies to cope with these situations, for example by highlighting negative consequences of use, and positive aspects of abstinence. These treatment approaches have demonstrated the ability to induce changes at the neurobiological level (43) and improve clinical outcomes (44, 45). Among the mechanisms underlying the impact of these interventions, coping strategies and the subject’s ability to anticipate and manage these at-risk situations have been identified as determinant factors (45–47).

Despite high prevalence in the general population, and the effectiveness of treatments, addiction is the psychiatric disorder with the most important “treatment gap,” i.e., the difference between the prevalence of a disorder and the number of individuals treated for that disorder (48). Indeed, the worldwide proportion of individuals with an addiction accessing treatment is estimated to be less than 25% overall, and less than 10% for alcohol and tobacco (49–52). This gap could be partially explained by a lack of insight, i.e., capability to recognize one’s mental illness, its symptoms and consequences, and to perceive the need for treatment (53, 54). Stigma associated with addiction, the desire to try to cope alone, but also the lack of knowledge about the treatment of addictive disorders could also impede access to treatment (55). This gap could also be explained by structural barriers, such as difficulties for some people to access standard treatment centers, e.g., rural areas, or time demands. However, delayed access to treatment could lead to an increase in addiction severity, associated with more complications, and poorer treatment outcomes (56).

Some of these barriers can be overcome by the use of mobile technologies. Mobile health (mHealth) offers new opportunities to improve prevention, screening, diagnosis and treatment of addictions (57). A number of people could benefit from screening and diagnostic tools easily accessible and anonymous, and smartphone apps could be designed for individual autonomous use, and reach large portions of the population who would otherwise not have access to a standard treatment. Ecological Momentary Intervention (EMI) is a treatment procedure characterized by the delivery of interventions, generally by messages on smartphones, in response to EMA self-reports in the app, to people as they go about their daily lives (ecological) and in the moment when it is most needed (momentary) (58, 59). For example, in addiction treatments, EMI apps may help to protect against relapse by prompting people to mobilize their coping resources in high-risk situations. Populations underserved or reluctant to face-to-face traditional treatment could benefit from such accessible technology (60, 61). For example, it has been demonstrated that a larger proportion of female, younger or older adults, or at-risk users access internet-based interventions than are typically seen in traditional treatment contexts (62–65). Thus, EMI presents cost efficient opportunities for treatments to be available to more people during times and in situations when they are most needed, without the need to consult a specialist (66).

A growing number of mobile medical interventions apps are being developed in all health areas (59, 67), including mental health (58, 68, 69). Several reviews have investigated apps targeting addictions [e.g., (70–72)], and some of them reported encouraging results for tobacco (72) and alcohol (73–76). Although fewer in number, apps targeting illegal substances also show promising results in reducing craving or use of cannabis (77–79), opioids (80), or various substances (81). However, the reduced number of available studies of high methodological quality or with sufficient statistical power (82), as well as heterogeneity in comparison conditions, outcomes or population tested, make it difficult to provide compelling evidence of efficacy upon abstinence or reduction of addictive use (69, 71), despite good feasibility and acceptability (68). This greatly limits for now their dissemination and adoption in the medical community (66, 83).

Nevertheless, these studies give us some indication for best outcomes. Among the major pitfalls frequently reported in mHealth apps (84, 85), difficulty to maintain engagement is important, and could be explained by design considerations, and user experience. For example, too frequent or too long EMA assessments can lead to user fatigue and discontinuation of use. On the contrary, simplicity, and easily accessible information have been identified to enhance user engagement (81, 86). Regular utilization is important because it contributes to the effectiveness of mHealth app, but, interestingly, could also be sustained by the user’s perception of effectiveness (87, 88). Tailored content and personalization could enhance the feeling of being concerned and consequently promote user engagement. Indeed, “one size fits all” interventions could miss the point, and addiction management has been shown to be more effective when treatments were personalized to target the specific needs and characteristics of each patient (89, 90). As previously mentioned, currently available research suggests that exposure to complex stimuli associated with using a substance/engaging in the addictive behavior, and linked to individuals’ unique history may represent a major predictor of relapse. The use of geolocation, for example, could contribute to detect, for each individual, the places most associated with addictive use (potentially increasing the risk of further relapse), and thus allow to define, in a personalized way, when to propose an intervention (91). In this line, Just-In-Time Adaptive Interventions (JITAIs) propose to adapt, improve and tailor the treatment delivery based on previous answers provided by the user, or information collected by the smartphone (92, 93).

Another crucial aspect for acceptability and dissemination of mHealth apps is their scientific and medical support (94, 95). This can be achieved in several different ways: by involving users and experts in the development of the app, but also by informing about the theoretical framework and/or evidence-based approaches on which the app and its content are based (85). For example, use of the Behavior Change techniques (BCTs) taxonomy (96) could help to describe active “ingredients” of the intervention (97) and could provide further exploration of its mechanisms of action in efficacy studies (98). For management of addictions, some BCTs have been reported as particularly efficient: “Avoidance/reducing of exposure to cues for behavior,” “Pros and cons”: listing and comparing the advantages and disadvantages of quitting, “Self-monitoring of behavior” as well as “Behavioral substitution”: engaging in alternative activities (99, 100).

In this context, we developed the Craving-Manager smartphone app targeting craving and personal situations at-risk for addictive use for people with addiction, whatever the addiction, substances and behaviors. This article describes the protocol for a multicenter randomized double-blind controlled trial with the primary objective of evaluating the efficacy of 4 weeks of use of the Craving-Manager app, as compared to a restricted version of the app (EMA only - placebo), among individuals on a waiting list for outpatient addiction treatment. We hypothesized that, in comparison to those receiving the restricted version of the app, those receiving the Craving-Manager app will be more likely to decrease the use of primary substance/addictive behavior over the 4 weeks use of the app.

## 2. Methods and analysis

### 2.1. Design

This study is a randomized double-blind controlled trial comparing two parallel groups. The intervention consists of 4 weeks of use of the Craving-Manager app. The control group will receive a restricted version of the app (EMA only). The study was registered on ClinicalTrials.gov (NCT04732676), and was approved by French Ethics Committee on 31 March 2021 (ID-RCB 2020-A01707-32).

### 2.2. Participants

Participants will be recruited among people requesting treatment for addiction in one of 6 specialized addiction treatment centers in France (Bordeaux, Bayonne, Limoges, Poitiers, Grenoble, La Réunion). Recruitment will take place before the beginning of standard treatment, while waiting for the first appointment. Eligible participants will be adults with at least one substance or behavioral addiction (including any addictive use/ behavior for which the participant feels a loss of control and/or that may lead to the need to seek addiction treatment*)*, who request help for that addiction, and with more than a 1-month-delay on the waiting-list before the first appointment. Participants should not have difficulty in understanding and writing French, and should be familiar with the use of a smartphone. Written consent will be collected after an informed consent procedure. Exclusion criteria concern people with somatic, cognitive or other disorders preventing the use of smartphone (deafness, impaired vision, illiteracy....), or people with medical, psychiatric or addictive disorders that warrants immediate treatment. People who are deprived of liberty due to an ongoing legal procedure, individuals under legal protection, under guardianship or curatorship, or not affiliate/ beneficiary of a social security scheme will not be included in the study in compliance with French Regulations on the participation of Humans in research.

### 2.3. Sample size calculation

A power analysis was performed to determine the sample size based on data from a previous EMA study among subjects beginning outpatient addiction treatment (101). We considered that a significant clinical effect was a decrease of at least 20% of addictive use frequency for 20% of the participants in the experimental group between the 1st and 4th week of smartphone app use. We expect that 5% of participants in the control group will present this decrease. To observe a difference of 15% (20% vs. 5%), with a 90% statistical power, a type 1 error rate of 5% and assuming 20% loss-to-follow-up (considered as failures), we need 137 participants randomized to each group (274 participants to be included in total).

### 2.4. Randomization and blinding

Participants will be randomized in two distinct groups: the Experimental Group will have access to the full active version of the Craving-Manager app (EMA and EMI) during 1 month, and the Control Group will have access to a restricted (placebo) smartphone app (EMA only) during 1 month.

Randomization will be done by the app, and will take place after confirmation of eligibility and the informed consent procedure. It will be done centrally in a 1:1 ratio, and stratified by inclusion center.

Participants will be unaware of the specific goals of the study or of the differences between the 2 versions of the app. The primary endpoint will be collected through EMA. Research staff will be unaware of group assignment and secondary endpoint collected through face-to-face interviews will be assessed blindly to the randomization group.

### 2.5. Measurements

Data will be collected through different questionnaires: the Addiction Severity Index (ASI), the Mini International Neuropsychiatric Interview (MINI), a Craving Evaluation scale, the Treatment Service Review (TSR), Feasibility and acceptability questionnaires, and some data will be directly collected by the smartphone app (see 2.7 Craving-Manager app).

The Addiction Severity Index (ASI) is a semi-structured interview to assess impairments that commonly occur due to substance-related disorders (102). The modified and validated French version of the ASI (m-ASI) will be used to take into account tobacco and addictive behaviors (103). The m-ASI explores six areas that may be affected by addiction: medical status, employment/support status, substance and behavioral addiction, family and social relationships, legal status, and psychological status.

The Treatment Service Review (TSR 6th version) is an inventory of the subject’s medical, psychosocial and psycho-educational contacts over the past 30 days (104).

The Mini International Neuropsychiatric Interview (MINI) is a structured diagnostic interview providing standardized assessment of major psychiatric disorders defined according to DSM criteria (105). For this study, we have adapted the French DSM-IV version of the MINI to meet DSM-5 criteria (6). Diagnoses of Substance Use Disorders (SUD), gambling disorder and internet gaming disorder will be explored with the DSM-5 criteria (with addition of the craving criterion as an exploratory measure for gambling and gaming). For other addictions (any other use/addictive behavior for which the participant feels a loss of control and/or that may lead to the need to seek addiction treatment), the DSM-5 diagnostic criteria for SUD will be adapted to the use/behavior concerned.

The Craving Evaluation scale, developed by the SANPSY lab at University of Bordeaux, explores craving for all substances and addictive behaviors reported by the participant: the frequency of craving, corresponding to the number of days craving was experienced during the past 30 days, as well as mean and maximum craving intensity on a numerical rating scale ranging from 0 (no craving) to 10 (extreme craving).

The Acceptability E-scale (AES) is a 6-item questionnaire that evaluates the extent to which participants find e-health systems acceptable. Each item is ranked on a 5-point Likert scale, generating a total score of acceptability ranging from 6 (lowest acceptability) to 30 (highest acceptability) (106).

The Digital Working Alliance Inventory (D-WAI) measures factors (goals, tasks and bond) to examine the therapeutic relationship between the participant and the app (107).

The user version of the Mobile Application Rating Scale (uMARS) is a simple tool that can be reliably used by end-users to assess the quality of mHealth apps (108). A translation has been published by the French National Health Agency (Haute Autorité de Santé) (109).

The Client Satisfaction Questionnaire (CSQ-8) is a multi-item measure of satisfaction related to healthcare (110, 111). The CSQ-8 total score ranges from 8 to 32. A higher score represents greater satisfaction.

Toxicological measurements will be systematically associated with the m-ASI, as previous research has found that the validity of self-report information on substance use is increased when biological assessments are included in the research protocol (112): Urinalysis (to detect opioids, methadone (EDDP), cocaine, benzodiazepines, cannabis and buprenorphine), alcohol and CO breath tests.

### 2.6. Study procedure and intervention

**Figure 1:**
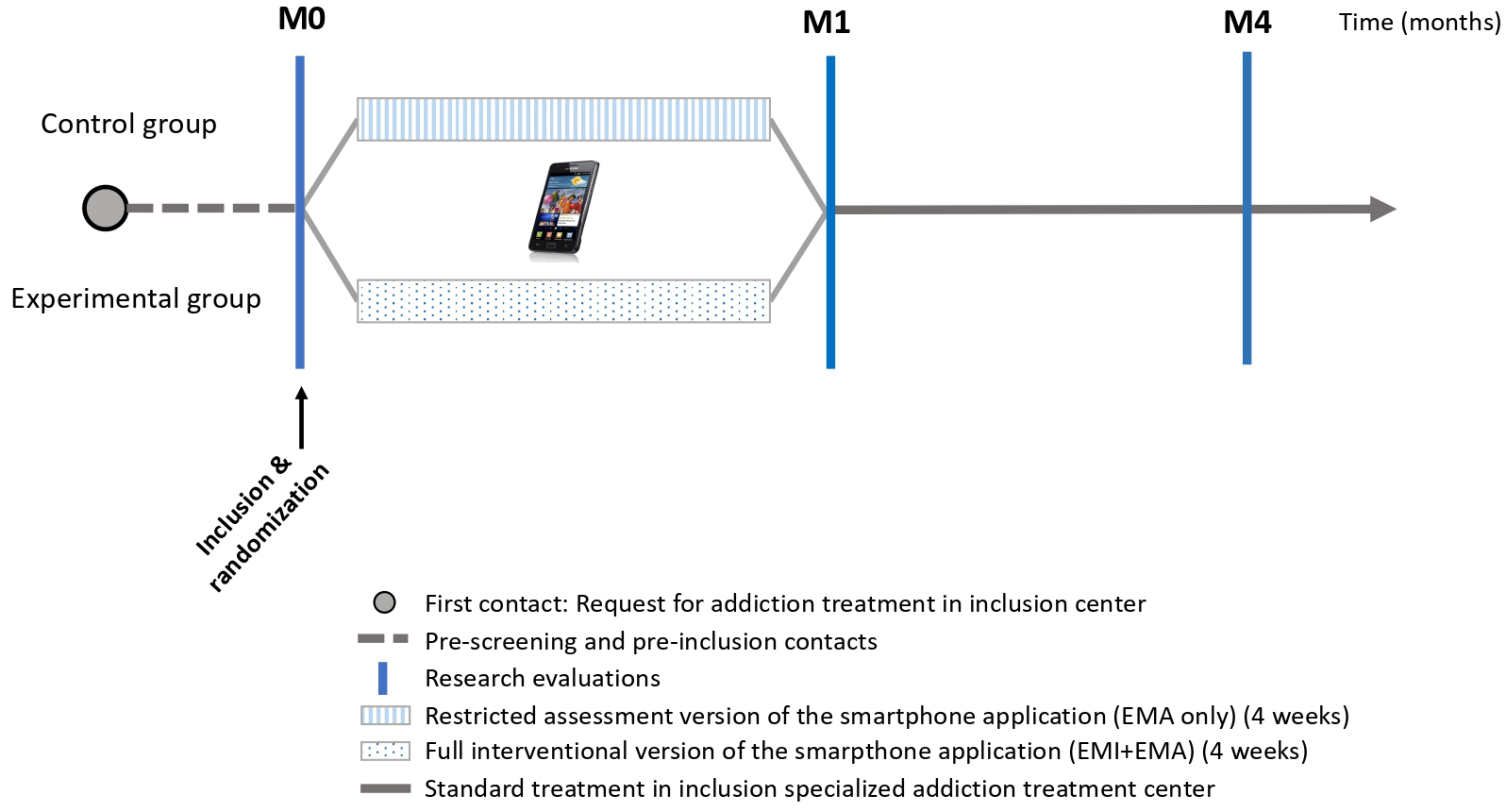
Description of study interventions and evaluations by group.

Participants will be recruited among patients on a waiting list for a first clinical appointment at the inclusion center, with at least 1-month delay before the first scheduled appointment (to avoid overlap of the intervention proposed by the Craving-Manager app with the intervention that will be provided later by the addiction clinic; Figure 1). First, a telephone screening interview will allow to explain the study and to check the main inclusion criteria. Then, interested and eligible participants will receive an inclusion visit (baseline) at the inclusion center to sign the informed consent, and receive a face-to-face research interview, with ASI, MINI, TSR and Craving Evaluation scale (see Table 1).

**Table 1:** Assessment procedure according to each visit.

	Screening	Baseline (M0)	Smartphone app	1-month Follow-up (M1)	4-month Follow-up (M4)
Criteria verification	x	x			
Informed consent		x			
Inclusion		x			
Randomization		x			
ASI		x		x	x
Craving scale		x		x	x
TSR		x		x	x
MINI		x			
Toxicological measurements		x		x	x
EMA			x		
EMI			x (for experimental group only)		
AES				x	
D-WAY				x	
uMARS				x	
CSQ-8				x	

They will then receive a smartphone loaned for the study, containing the app. The randomization will be performed by the app, and will allow to unlock one of the 2 versions of the app: a restricted version (EMA only–placebo) for the Control Group, and a full active version of the Craving-Manager app (EMA and EMI) for the Experimental Group (see below). The use of a restricted version of the app in the Control group is intended to control for potential increase in empowerment or insight as a result of answering self-questionnaires. Indeed, previous studies have documented the positive impact of the self-monitoring on substance use reduction in EMA protocols (113).

All participants will be trained to use the smartphone. As the Craving-Manager app has been designed to be used as a stand-alone app, several tutorials are provided while the app is being used. The completion of the app questionnaires is done in autonomy by the participant, but a research assistant (dedicated to the technical aspects only and not involved in participant assessment, to maintain the blindness) is available to answer any questions and provide help, if needed. Participants from both groups will receive training, and they will not be aware of the differences between the 2 versions of the app.

The app will be active for 4 consecutive weeks and then it will be automatically deactivated. At the one-month follow-up, the smartphone will be returned and all participants will receive a face-to-face research interview evaluation with ASI, Craving Evaluation scale, and feasibility, acceptability and Intervention Satisfaction scales (AES, D-WAI, uMARS, CSQ-8).

At the 4-month follow-up, participants will receive a face-to-face research interview evaluation with ASI, TSR and Craving Evaluation scale. Financial compensation will be provided as a function of visits completed (with a maximum of 120 euros for full participation), but regardless of frequency of app use, to avoid influencing completion rate.

### 2.7. Craving-Manager app

The Craving-Manager app was developed by SANPSY lab at University of Bordeaux, Bordeaux, France. According to the Cue-Craving-Use model of addiction proposed on the basis of previous EMA research (7, 37), this trans-addiction app was designed to anticipate, monitor and help the participants to cope with cues, craving and addictive use, whatever the substance or the behavioral addiction. The app covers all the addictions already (or waiting to be) acknowledged by the DSM (including tobacco, alcohol, cannabis, opiates, cocaine, crack, stimulants, sedatives, gambling, gaming) but also covers other potential addictions, including food, shopping/spending, exercise, work, sex/pornography, trading, smartphone/SMS, Social network/chat, television, online dating, stealing/ kleptomania, and an open-response item has been added for any other use/addictive behavior for which the participant feels a loss of control/ need for treatment. Interventions (see Table 2 below) are derived from standard approaches of addiction treatment, mainly Relapse Prevention (114), CBT (42) and to a lesser extent mindfulness (115). In order to improve compliance/acceptability, usability was evaluated among a sample of end-users and clinicians on a first version of the app, and changes in the functionality of the app were made on an as-needed basis.

**Table 2:** Types and number of interventions proposed by the Craving-Manager app according to the Behavior Change Techniques taxonomy (96).

Code	Type of intervention according to the BCT taxonomy	Number of interventions
1.2	Problem solving/ coping planning	6
1.3	Goal setting (outcome)	1
1.4	Action planning	1
1.7	Review of outcome goals	1
2.2	Feedback on behavior	1
2.3	Self-monitoring of behavior	6
3.1	Social support (general)	1
3.3	Social support (emotional)	1
4.1	Instruction on how to perform a behavior	1
4.3	Reattribution	2
9.2	Pros and cons	3
11.2	Regulation of negative emotions	2
11.3	Conserving mental resources	2
12.3	Antecedents	3
12.4	Distraction	1
15.1	Verbal persuasion to boost self-efficacy	2

The app begins with an initial questionnaire for systematic assessment of DSM-5 diagnosis for all substance and behavioral addictions (6). The app then collects the primary (i.e., main problematic) addiction(s), on which the app will be focused, according to participant’s demand and current diagnosis. In the full app version dedicated to the Experimental Group, person-specific cues are also explored (37) for further personalization of EMA questionnaires, and at-risk locations, i.e., previously associated with addictive use or craving, are identified on a map. In this full app version, supportive/protective factors (such as family/friends or pleasant activities to distract from craving,) but also participant perceived disadvantages of continued use, and on the contrary, participant perceived benefits of quitting, are collected for personalization of further support/intervention EMI messages. Participants receiving the full version of the app will also receive informational contents about the definition of addiction and craving, delivered by an Embodied Conversational Agent (ECA) (101, 116, 117).

During the 4-week use of the app, participants from both groups are prompted with 4 daily EMA questionnaires (5 min each), randomized across the day in a time slot of 14 h. Electronic interviews assess the presence of cues, craving or addictive use, since the previous assessment, for the primary addiction(s), as well as a list of mood states, and use of other substances or addictive behaviors. In the Experimental group only, an EMI is immediately proposed to participants who report exposure to cues, craving, or addictive use (Table 3). In this group, additional EMI is also proposed on demand, or when the app detects that the participant is approaching a place previously marked as at-risk on the map, thanks to continuous geolocation collected at a rate of 0.2 Hz. Each time cues, craving or addictive use are reported, the participant has the possibility to add new cues/at-risk locations or situations in the registered list for further monitoring.

**Table 3:** Framework’s resume of the Craving-Manager app functioning.

Situation	Decision rules	Decision point	Tailoring variables	Intervention options (IO)* according to BCT Taxonomy (see Table 2)
Addictive use report	IF use = TRUE since the previous assessment AND participant consents THEN see IO column.	Daily EMA prompt	Addictive use Type of substance use/addictive behavior Participant acceptance	1.2/1.3/ 2.3 + 12.3 /3.3/ 4.3 + 1.4/ 9.2/11.3 + 11.2 /12.4 /15.1
Craving report	IF level of craving is ≥4 (out of 7) OR IF level of craving is > last week’s mean level of craving AND participant consents THEN see IO column	Daily EMA prompt	Level of craving Type of substance use/addictive behavior Participant acceptance	1.2/2.3 + 12.3/2.3 + 1.2/3.3/4.1/9.2/11.3 + 11.2 /12.4
Cues report	IF exposition to a cue = TRUE AND participant consents THEN see IO column	Daily EMA prompt	Being exposed to a cue Participant acceptance	1.2/2.3 + 1.2/3.3 /11.3 + 11.2/12.4
At-risk location detected (GPS)	IF distance < fixed distance (10–90 meters) around one of the at-risk location points AND IF participant consents THEN see IO column.	Anytime, when detected	Distance to at-risk location Participant acceptance	1.2/2.3 + 1.2/3.3 /11.3 + 11.2 /12.4
On-demand help request	IF click on the help button AND report: risk of use, use, craving, exposition to a cue THEN see IO column.	Anytime, on participant’s demand	Individual’s need Type of situation reported	1.2/1.3/2.3 + 1.2/2.3 + 12.3/3.3/4.1/4.3 + 1.4 9.2/11.3 + 11.2/12.4/15.1

*When several options are possible, the intervention is drawn at random, using a draw without discount rule, to maximize alternation of intervention options, and avoid fatigue effects.

A list of types of intervention proposed by the app is described in Table 2 according to the Behavior Change Techniques (BCT) taxonomy (96). Among the Craving-Manager interventions, the most frequent types are Problem solving/ coping planning [1.2] interventions that aim to help to manage situations that could lead to addictive use, Feedback and Self-monitoring [2.2–2.3] interventions that aim to guide user to be more aware and focused on his/her environment and its triggers to some problematic behaviors, Pros and cons [9.2] interventions that are reminders regarding negatives consequences of addictive use and benefits from quitting, and Antecedents [12.3] interventions suggest ways to avoid cue exposure drawing from past experiences.

At the end of each week, participants in the Experimental group receive a personalized feedback about cues encountered, craving and addictive use across the past 7 days, and motivational messages to encourage effort to reduce/quit primary addiction(s). Attention is also drawn to other substances/addictive behaviors, in order to avoid the risk of switching addiction (118). The app also monitors evolution of self-efficacy, and presents a graph of EMA response rates to encourage completion. The control group only receives feedback on the EMA completion rate.

The functioning of the app is described in Table 3 according the Nahum-Shani and colleagues list of contents and design principles of JITAI (92). *Decision points* are the moment when the choice of an intervention can be done. *Decision rules* determines whether or not an intervention is proposed, and which one, among *Intervention Options* (IO), thanks to an examination of *Tailoring variables*.

### 2.8. Analysis strategy

The primary objective to evaluate the efficacy of the Craving-Manager app over 4 weeks, as compared to a restricted (placebo) version of the app, will be assessed by comparing the percentage of participants for whom a 20% decrease in frequency of use of primary substance/ addictive behavior was observed, between the 1st and 4th week of smartphone app use, in the experimental group versus the control group. This 20% reduction threshold was defined in line with decrease in the “percentage of using days” observed with smartphone apps targeting alcohol (75) and substance use disorders (80).

Analyses will be conducted according to the intent to treat principle, in which all randomized participants will be included in the group in which they were first randomized and all their data will be used, regardless of the changes over the study duration.

In addition, in case some participants stopped using the app before the expected termination, a sensitivity analysis will be performed using the LOCF (Last Observation Carried Forward) approach, to calculate the decrease in frequency of use of primary substance/behavior not on the 4^th^ week, but on the last 7 days with at least one EMA questionnaire completed.

The comparison between groups will focus on the proportion of participants that reach a 20% decrease in use of primary substance(s)/ addictive behavior(s). This is a superiority trial (bilateral test). The 95% confidence intervals (exact binomial distribution) in each group will be calculated. These 2 proportions will be compared with a Chi2 test or with Fisher’s exact test, according to the size of the expected values under the hypothesis of independence. Mixed effects logistic regression model will be used to adjust stratification factor (random effect on centers) and other major confounding factors. Assumption of the models (log-linearity of the associations) will be systematically checked.

Efficacy of the intervention will also be examined on secondary endpoints: Multifactorial addiction severity (assessed with ASI Composite Score for the primary addiction) at 1-month and 4-month follow-ups, and craving self-reported in the last EMA week, and reported at 1-month and 4-month follow-ups in the Craving Evaluation Scale.

Further analysis will explore factors associated with efficacy, among initial severity of addiction (ASI Composite Score for the primary addiction at inclusion), initial craving frequency and intensity (with Craving Evaluation scale), type of addiction, comorbid psychiatric disorders, and past or concomitant addiction treatment. Temporal evolution of craving and substance use/addictive behaviors over the 4 weeks of app use will be described.

Feasibility, acceptability and intervention satisfaction will be assessed at the one-month follow-up by the number of days and frequency of use of the app (calculated from EMA responses: number of questionnaires answered on total number of questionnaires proposed by the app), with acceptability questionnaires (AES, uMARS and D-WAI) and with the specific CSQ-8 questionnaire assessing intervention satisfaction, rate of participants who did not finish the study, and reasons for stopping.

Impact of the 4 weeks use of the app on subsequent treatment will be assessed at 4-month follow-up with the rate of first-time attendance to the standard treatment, time to first-time attendance to the standard treatment, compliance with standard treatment (TSR), and evolution of severity of addiction during the standard treatment (ASI Composite Score).

Reliability of the app to diagnose addictive disorders will be examined by comparison with diagnosis explored at inclusion with MINI (human interview).

The quantitative variables will be compared by Student t test if the conditions of validity are respected (normal distribution, homogeneous variances). If the variances are different between both groups, we will use Student t test for unequal variances; if the distribution is not normal, we will use non-parametric Wilcoxon test. Mixed effects linear regression models will be used to adjust on stratification factor (random effect on centers) and other major confounding factors. Assumptions of the models (normal distribution, homogeneous variances, linearity of the associations) will be systematically checked. In some cases, it may be necessary to transform or recode some variables to adapt them to the conditions under which statistical tests are applied.

Statistical analysis will be performed using JMP Pro 16.0 (SAS Institute, Cary, North Carolina), and HLM software 8.0 (Scientific Software International) for mixed effects linear regression models. For all the tests, the level of significance will be set at *p* < 0.05.

## 3. Discussion

This article describes the protocol for a multicentered randomized double-blind controlled trial with the primary objective to evaluate efficacy of using the Craving-Manager app over 4 weeks, as compared to a restricted, non-interventional (placebo) version of the app, among individuals on a waiting list for outpatient addiction treatment. To our knowledge, this study is the first to test an mHealth trans-addiction app designed to monitor and manage craving, based on evidence-based interventional approaches.

The anticipated strengths of this study are the randomized double-blind controlled design which offers the opportunity to determine, in a stringent way, the potential impact of the intervention (using the Craving-Manager app) on the outcome (decreased use of primary substance/addictive behavior) over the 4 weeks of app use. A restricted EMA version of the app will be used in the Control group as a placebo to control for the potential effect (on empowerment and insight) of self-monitoring on substance use (113). An important strength of the app is to propose a high level of personalization. For example, the choice of the primary main addiction(s) targeted by the app is based on both current DSM-5 diagnosis and self-report demands of the user. Personalization is also achieved through monitoring of a large range of person-specific cues, Interventional contents adapted on individual experiences for protective factors, List of disadvantages of continued use and benefits of quitting, and Possibility to add new cues encountered in daily life during the study.

An important limitation to acknowledge is that the Craving-Manager app relies on the capacity of the participant to be able to be aware of his/her craving and in capacity to report it, which implies some significant insight capacity. However, participants will be recruited among people requesting addiction treatment, which will ensure a sufficient level of clinical insight. Moreover, through information contents (definition of craving), and regular assessments of situations preceding substance use/addictive behavior, the Craving-Manager app may help to improve the insight of craving. In this study, there is no biological monitoring of addictive use and no physiological measures related to craving reactivity. Further studies should explore passive monitoring of craving/relapse risk to increase the ability of the app to detect when an intervention is needed, and to limit burden associated with self-reports.

To conclude, if proven effective in reducing use of substance/addictive behaviors, the Craving-Manager app will offer a new stand-alone mHealth solution to autonomously diagnose and manage addiction in daily life. Based on the huge estimated proportion of individuals with an addiction who are not treated (49–52), this app could give access to treatment to a large number of people, and thus participate to reducing the global burden of disease, both by reducing addictive use and its harmful consequences, but also by accelerating access to treatment at a lower stage of severity, thus increasing chances of success. Such mHealth solutions, user-friendly and accessible, could be particularly relevant for individuals with low severity, or in the “pre-addiction” stage (119), as it could represent a way to enable individuals to increase their awareness of their symptoms and the need to receive treatment. The Craving-Manager app could also facilitate access to outpatients or inpatients addiction treatments for the most severe patients, by reducing the potential self-stigma associated with their disorder. The Craving-Manager app could also be of interest as an add-on to traditional addiction treatments, offering a daily support between two treatment visits, but also feedback for the clinicians on the patient’s evolution, and alert on the occurrence of a possible relapse.

## Ethics statement

The studies involving human participants were reviewed and approved by French Ethics Committee for the Protection of Persons (CPP SUD MÉDITERRANÉE V). The patients/participants provided their written informed consent to participate in this study.

## Author contributions

FS, SM, LDo, HG, and MA wrote the manuscript. FS and MA wrote specifications of the app and conceived and designed the study. FS, SM, LDo, HG, and CF tested the app. LDo, CF, J-MA, LDu, and PP provided significant input on design and/or contents of the app. YL developed the app. ES developed the embodied conversational agent. MA is the principal investigator in charge of the study. All authors contributed to the article and approved the submitted version.

## Craving-Manager RCT investigator group

Members of the Craving-Manager RCT investigator group Marc Auriacombe (CH C. Perrens, Bordeaux, France), Jean-Pierre Daulouède (BIZIA, Bayonne, France), Maurice Dematteis (CHU Grenoble-Alpes, Grenoble, France), Nemat Jaafari (CH H. Laborit, Poitiers, France), David Mété (CHU F. Guyon, La Réunion, France), and Philippe Nubukpo (CH Esquirol, Limoges, France).

## Funding

This project was supported by the national DGOS grant PHRC-N 2018–0528 (MA, Centre Hospitalier Charles Perrens), by office and staff support from Centre Hospitalier Charles Perrens (CHCP), and by Internal funds from Université de Bordeaux.

## Conflict of interest

The authors declare that the research was conducted in the absence of any commercial or financial relationships that could be construed as a potential conflict of interest.

## Publisher’s note

All claims expressed in this article are solely those of the authors and do not necessarily represent those of their affiliated organizations, or those of the publisher, the editors and the reviewers. Any product that may be evaluated in this article, or claim that may be made by its manufacturer, is not guaranteed or endorsed by the publisher.
